# The Influence of Fluidic Flow Stress on the Development of the Secondary Palate

**DOI:** 10.3390/jdb14010009

**Published:** 2026-02-12

**Authors:** Masayo Nagata, Satoru Hayano, Ziyi Wang, Takahiro Kosami, Hiroshi Kamioka

**Affiliations:** 1 Department of Orthodontics, Okayama University Hospital, Okayama 700-8525, Japan; p6ju0rxh@s.okayama-u.ac.jp; 2 Department of Molecular Biology and Biochemistry, Graduate School of Medicine, Dentistry and Pharmaceutical Sciences, Okayama University, Okayama 700-8525, Japan; 3 Department of Orthodontics, Graduate School of Medicine, Dentistry and Pharmaceutical Sciences, Okayama University, Okayama 700-8525, Japan; de427020@s.okayama-u.ac.jp (T.K.); kamioka@md.okayama-u.ac.jp (H.K.)

**Keywords:** mechanical stress, palatal development, β-catenin, YAP

## Abstract

Craniofacial development is orchestrated by a finely regulated interplay of numerous genes and signaling pathways. Palatogenesis proceeds through a complex, stepwise process, in which endogenous mechanical stresses within tissues have been implicated. However, the impact of exogenous fluidic flow mechanical stress derived from maternal movement on palatal development remains unclear. In this study, we investigated the effect of exogenous fluidic flow mechanical stress on palatal morphogenesis, focusing on the horizontal outgrowth of palatal shelves after elevation. Palatal tissues dissected from mouse embryos were subjected to organ culture with or without mechanical loading (loaded and unloaded groups, respectively). Stress magnitude was quantified by calculating wave energy, and morphometric and molecular analyses were performed. Compared with the unloaded group, palatal shelves in the loaded group showed significant increases in thickness and volume, accompanied by enhanced cell proliferation, nuclear translocation of YAP and β-catenin, and upregulation of the osteogenic markers Osterix and Osteocalcin. No significant difference in apoptosis was observed. These findings indicate that exogenous mechanical stress promotes cell proliferation and osteogenic differentiation through the Hippo and WNT/β-catenin pathways in palate explants. Our results suggest that moderate maternal movement-induced mechanical stress contributes to normal palatogenesis, providing new insights into the mechanisms underlying cleft palate.

## 1. Introduction

Craniofacial development involves the intricate temporal and spatial regulation of numerous genes and their transcripts. Disruptions in this harmonious gene expression, caused by various external stresses, genetic mutations, or teratogenic agents, can lead to congenital malformations. Cleft lip and palate is one of the most common craniofacial birth defects [1].

The palate is formed through a complex series of events including vertical growth of the palatal shelves, elevation above the tongue, medial extension, fusion at the midline, and subsequent three-dimensional growth [2]. Cleft palate results from the failure of palatal shelves to fuse [3]. Palatal development is controlled by a complex series of signaling pathways. Transforming growth factor beta (TGF-β) has long been known to play a crucial role in palatal shelf fusion [4]. TGF-β3, in particular, has been reported to be involved in the epithelial breakdown at the site of palatal shelf fusion [5]. β-catenin, a key regulator of cell proliferation, has been shown to regulate TGF-β3 expression and thus contribute to palatal shelf fusion [6]. Sonic hedgehog (Shh) positively regulates fibroblast growth factor 10 (Fgf10) expression via a positive feedback loop, thereby controlling cell proliferation and palatal shelf elongation [7]. Recently, in addition to these biochemical signaling pathways, increasing attention has been paid to the role of mechanical forces in palatal development. Endogenous mechanical stimuli have been shown to play important roles in the morphogenesis of craniofacial tissues, including the palate [8,9]. In contrast, exogenous mechanical stimuli have been reported to contribute to skeletal formation and bone mineralization in the joints of mice and chickens [10,11]. However, the effects of exogenous mechanical stress on palatal development in mice have not yet been reported.

In addition to genetic mutations, environmental factors have been implicated in the failure of palatal shelves to fuse [12]. Pregnancies involving mothers with severe disabilities are associated with a higher incidence of low birth weight infants [13], and low birth weight infants have a higher incidence of cleft lip and palate [14]. Considering that fetuses of mothers with severe disabilities may experience less mechanical stress during pregnancy, it is hypothesized that moderate mechanical stress generated by maternal exercise plays a crucial role in the development of organs and tissues throughout the body, including normal palatal formation.

One of the signaling pathways involved in the transmission of mechanical stress is the Hippo pathway. The Hippo pathway regulates various biological processes, such as cell differentiation, organ size control, and regeneration, through the nuclear translocation of yes-associated protein/transcriptional co-activator with PDZ-binding motif (YAP/TAZ) [15]. Nuclear translocation of YAP/TAZ requires its dephosphorylation, while excessive phosphorylation leads to ubiquitination and degradation, limiting its abundance [16]. Integrins, a type of extracellular matrix protein, receive mechanical stress through cell adhesion mechanisms and regulate YAP/TAZ activation, mediating the transmission of mechanical stress into cells [17].

Numerous studies have demonstrated the involvement of mechanical stress in tissue formation. In craniofacial development, mechanical stress generated by differences in cell proliferation rates during tooth germ formation has been shown to regulate tooth germ morphology [18]. Additionally, mechanical stress exerted on the skull by brain growth has been reported to promote bone formation at the cranial sutures [19]. In the palate, endogenous mechanical stress generated by hyaluronic acid accumulation, extracellular matrix remodeling by YAP/TAZ, and actomyosin contraction has been suggested to contribute to the vertical extension of the palatal shelves [20]. Furthermore, it has been reported that endogenous mechanical stress generated by actomyosin contraction and regulation of Piezo ion channels may be involved in the fusion of the left and right palatal shelves [21]. However, these studies have focused on endogenous mechanical stress generated within the fetal palatal tissue, and the detailed effects of exogenous mechanical stress generated by maternal exercise on palatal development remain unclear. To date, how continuous exogenous mechanical stimulation, such as maternal movement, affects fetal palatal development, and which intracellular signaling pathways mediate these effects, has not been experimentally investigated. The originality of this study lies in addressing this unexplored area. In this study, the magnitude of exogenous mechanical stress was set within a physiologically plausible range that, when converted into equivalent maternal physical activity, corresponds to moderate exercise. We aimed to clarify the effects and underlying molecular mechanisms of exogenous mechanical stress on palatal development. Therefore, this study aimed to elucidate the effects of mechanical stress on palatal development by experimentally reproducing exogenous mechanical stress caused by maternal exercise, focusing on the horizontal growth of palatal shelves after their elevation during palatal development.

## 2. Materials and Methods

### 2.1. Organ Culture of Palatal Tissue and Measurement of Mechanical Stress

**Figure 1:**
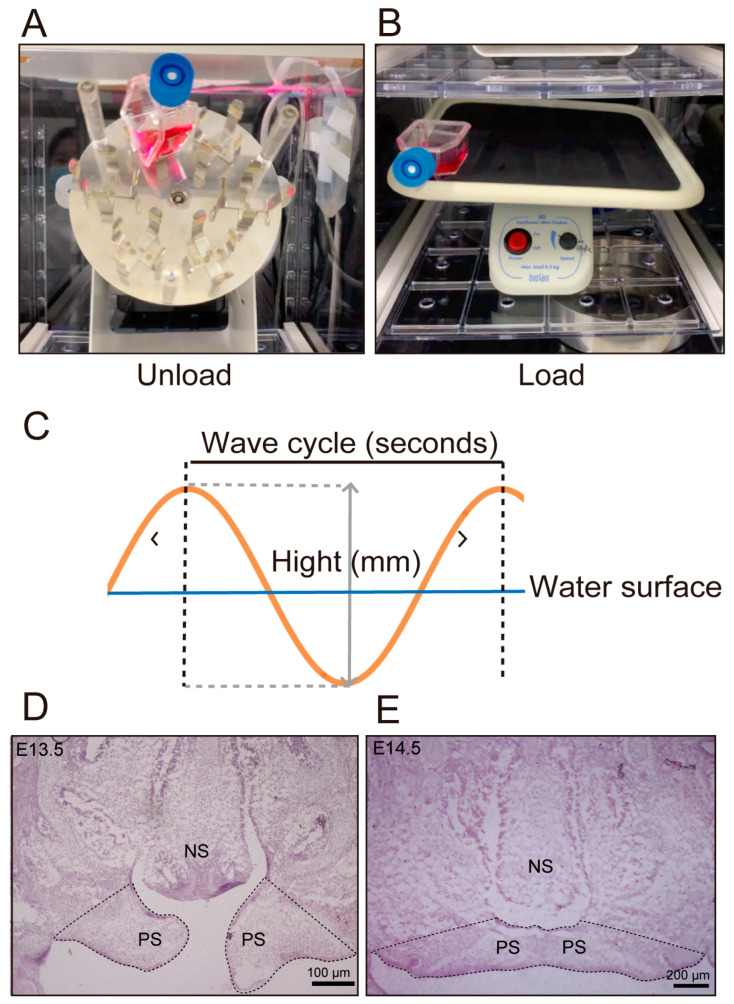
Organ Culture and Measurement Methods. (**A**,**B**) Organ culture apparatus. Unloaded group (without mechanical stress) was cultured in a rotary incubator (**A**), while the loaded group (with mechanical stress) was cultured in a shaker (**B**), both for 24 h. (**C**) Method for evaluating applied mechanical stress. Measurements were taken based on the height and period of waves appearing in the culture medium. (**D**,**E**) Frontal section of the palate in an E13.5 (**D**) and E14.5 (**E**) mouse embryo (H-E staining). The dotted area in the figure was used as a comparison region to compare the number of reactive cells (NS: nasal septum, PS: palatal process).

Pregnant ICR mice (CLEA, Tokyo, Japan) were euthanized by carbon dioxide inhalation followed by decapitation. Palatal tissues from 13.5- and 14.5-day-old mouse embryos were obtained after decapitation of the embryos and used for tissue culture and histological analysis. A total of 17 embryos at 13.5 days and 18 embryos at 14.5 days were used. The collected tissues were cultured for 24 h under a humidified atmosphere (37 °C, 95% oxygen, 5% carbon dioxide) according to a previously reported method [22]. The tissues were divided into an unloaded group, which did not receive mechanical stress, and a loaded group, which received mechanical stress. Samples were assigned to each group in a random manner, without pre-selection based on palatal morphology. Prior to establishing the current ex vivo organ culture system, we attempted whole-head ex vivo culture, including the mandible and tongue, to better mimic the in vivo environment. However, in these preliminary experiments, the deep regions of the tissue were not sufficiently perfused by the culture medium, resulting in tissue necrosis. Therefore, we conducted organ culture using only the region from the cranial base to the maxilla of the mouse embryo. In the usual culture, a rotary shaker (Rotary mixer NRC-200, Nissin, Shinonome, Hiroshima, Japan) (Figure 1A) was used for air circulation in the culture medium and suspension of the cultured tissue. However, in this study, the unloaded group was cultured using a rotary shaker as usual, while the loaded group was cultured using a shaker (vibration frequency: 1 cycle/s, Sunflower mini-shaker, Funakoshi, Hongo, Tokyo, Japan) (Figure 1B). The culture was performed in a 25 mL tissue culture flask (Tissue Culture Flask Canted Neck Blue Vented Cap; Corning Incorporated, Corning, NY, USA) containing palatal tissue and 10 mL of BGJb medium (Cell Signaling Technology, Danvers, MA, USA). To evaluate the intensity of shaking, the height of the waves generated on the surface of the culture medium and the number of waves counted per minute were measured. The mechanical stress applied to the tissue was calculated as wave energy based on the calculated wave height and period (Figure 1C). Wave energy (P) was calculated using the formula P = pg2H2T/32π (P: wave energy (W/s), p: specific gravity, g: gravitational acceleration, H: wave height (m), T: period (s)), where p = 1 and g = 9.8 (m/s^2^) [23]. Furthermore, since W = 1/4.184s (cal), the wave energy was converted to calories and then converted to the energy consumed by humans, assuming that 3.0 kcal was consumed per minute of walking, to evaluate the magnitude of mechanical stress.

### 2.2. Micro-CT Imaging

After 24 h of organ culture, embryonic mouse heads at embryonic day 14.5 were subjected to micro-CT imaging to compare palatal volumes between the unloaded and loaded groups. Since the target tissues in this study were soft tissues, samples were stained with a contrast agent according to the manufacturer’s protocol to enhance image clarity. Specifically, tissues fixed in 4% paraformaldehyde solution diluted in phosphate-buffered saline (PBS; Thermo) (Nacalai Tesque, Kyoto, Japan) were immersed in a solution containing 1% phosphotungstic acid (Thermo Scientific, Waltham, MA, USA) diluted in purified water and 7% ethanol, and stained at room temperature for 24 h in 15 mL tubes. After staining, the samples were rinsed in running water, stored in 7% ethanol, and imaged using a micro-CT system (Skyscan 1174, Bruker, Kontich, Belgium). Imaging was performed under the following conditions: pixel size = 12.9 μm, peak voltage = 50 kV, peak current = 800 μA, with a 0.5 mm aluminum filter. For image analysis, SkyScan software (NRecon v.1.6.4.8 and CT Analyser v.1.16.1.0, Bruker) was used. In both the unloaded and loaded groups, a rectangular region of interest (ROI) of 0.1 mm^2^ was defined in the coronal plane, starting 1.1 mm posterior to the first appearance of the secondary palate. A total of 88 consecutive images were analyzed. Three-dimensional reconstruction was performed using CT Analyser, and the X-ray–opaque area within the ROI of each of the 88 slices was quantified. The summed value of these areas was used as the volume index, representing the relative volume of the palatal shelves. These volume indices were then compared between the unloaded and loaded groups.

### 2.3. Immunohistochemical Analysis

To compare the effects of mechanical stress loaded on palatal tissue with respect to cell proliferation and the expression of osteoblast markers, which are suggested to be associated with the classical Wnt/β-catenin pathway [24], immunohistochemical analyses were performed using phospho-histone H3 and Osterix. After organ culture, the tissues were fixed with 4% paraformaldehyde diluted in phosphate-buffered saline (PBS) according to the method of Kawamoto et al. [25], followed by sucrose substitution and cryo-embedding using Tissue-Tek O.C.T compound (Sakura Finetek, Torrance, CA, USA). Serial cryosections of 6.0 μm thickness were prepared in the frontal plane using the film method [26] with a cryostat (CM3050S, Leica, Tokyo, Japan), beginning 500 μm posterior to the incisive foramen. For each specimen, twelve consecutive sections were obtained to ensure that equivalent anatomical regions were analyzed across all samples. Randomly selected sections (the number of sections analyzed is indicated in each figure legend) were permeabilized with 0.3% Triton X diluted in PBS, post-fixed with 4% paraformaldehyde in PBS, and blocked with Blocking One Histo (Nacalai Tesque) for 10 min. Primary antibodies were incubated overnight at 4 °C, followed by secondary antibodies for 1 h at room temperature. For nuclear staining, Hoechst^®^ 33342 (Thermo Scientific, #33342) was applied for 10 min. The sections were then mounted onto glass slides (Matsunami, Osaka, Japan), covered with Dako Fluorescence Mounting Medium (Agilent, San Diego, CA, USA), and sealed with cover glasses (Matsunami). Imaging was performed using a confocal laser microscope (LSM780, Carl Zeiss Promenade, Jena, Germany). Fluorescence images were analyzed with ImageJ (version 1.53k NIH, Bethesda, MD, USA) to quantify the total number of cells in the defined comparison area. Positive cells were identified visually based on distinct staining and counted. The comparison area was defined as the region between a straight line connecting the nasal-side corner of the palatal shelf and the labial groove ridge, extending to the tip of the palatal shelf (Figure 1D,E). The following antibodies were used: primary antibodies—E-cadherin (Invitrogen, Waltham, MA, USA, #14-3249-82), phospho-histone H3 (Abcam, Cambridge, UK, #ab1791), and Osterix (Abcam, #ab209484); secondary antibodies—Alexa Fluor^®^ 488 goat anti-rabbit IgG (Invitrogen, #A11008) and Alexa Fluor^®^ 594 goat anti-mouse IgG (Invitrogen, #A11005).

### 2.4. TUNEL Assay

Frozen sections prepared from the same region as for immunohistochemical analysis were randomly selected. After post-fixation with 4% paraformaldehyde solution diluted in PBS at room temperature for 20 min, cell death was evaluated using the TUNEL In Situ Cell Death Detection Kit, Fluorescein (Roche Applied Bioscience, Basel, Switzerland), according to the manufacturer’s instructions. For nuclear staining, sections were incubated with Hoechst^®^ at room temperature for 10 min. Imaging was performed with a confocal laser microscope (LSM780, Carl Zeiss, Jena, Germany), and the comparison area was defined in the same manner as in the immunohistochemical analysis. Fluorescence images were analyzed with ImageJ (NIH, Bethesda, MD, USA) to quantify the number of TUNEL-positive cells within the defined region (Figure 1D).

### 2.5. Quantitative RT-PCR

Total RNA was extracted using the RNeasy Mini Kit (Qiagen, Hilden, Germany) according to the manufacturer’s protocol. Reverse transcription was performed with the ReverTra Ace^®^ qPCR RT Kit (TOYOBO, Osaka, Japan). The resulting complementary DNA (cDNA) was analyzed via quantitative real-time polymerase chain reaction (qPCR) using gene-specific primers and the SYBR Green Real-time PCR Master Mix (TOYOBO). Relative expression levels of PCR products were quantified with the LightCycler^®^ System (Roche Diagnostics, Mannheim, Germany). The sequences of the gene-specific primers used in this study are listed in Table 1.

### 2.6. Western Blot Analysis

To examine the effects of mechanical stress on palatal tissue on YAP expression and the expression of β-catenin, which has been reported to be involved in palatal development [27], Western blot analysis was performed to compare YAP, β-catenin, and phospho-β-catenin expression in the loaded and unloaded groups. Samples were divided into cytoplasmic and nuclear fractions for each group. After culture, palatal tissues were washed with PBS and proteins were collected using NE-PER Nuclear and Cytoplasmic Extraction Reagents (Thermo Fisher Scientific, Waltham, MA, USA). Protein concentrations were quantified using the BCA Protein Assay Kit (Thermo Fisher Scientific) and adjusted to 1.0 μg/μL for the cytoplasm and 0.5 μg/μL for the nucleus. Each sample was separated by SDS-PAGE and transferred to a polyvinylidene difluoride (PVDF) membrane. The transferred membrane was blocked with 5% skim milk in TBS-T for 60 min at 4 °C and incubated overnight with primary antibodies: β-catenin (Cell Signaling Technology, #D10A8), phospho-β-catenin Ser552 (Cell Signaling Technology, #D8E11), and YAP (Santa Cruz Biotechnology, Santa Cruz, CA, USA, sc-271134, #B2720). As loading controls, GAPDH (Cell Signaling Technology, #2118) and Lamin B1 (Santa Cruz Biotechnology, #K1720) were used. After overnight incubation, the membrane was washed thoroughly and incubated with secondary antibody, Anti-rabbit IgG, HRP-linked Antibody (Cell Signaling Technology, #7074) for 1 h at room temperature. Bound antibodies were detected using a 20X LumiGLOR.

### 2.7. Statistical Analysis

Data from each experimental group are expressed as mean ± standard deviation (SD). Unpaired *t*-tests were used to compare the two groups. Differences were considered significant when *p* < 0.05.

## 3. Results

### 3.1. Quantification of Mechanical Stress

**Figure 2:**
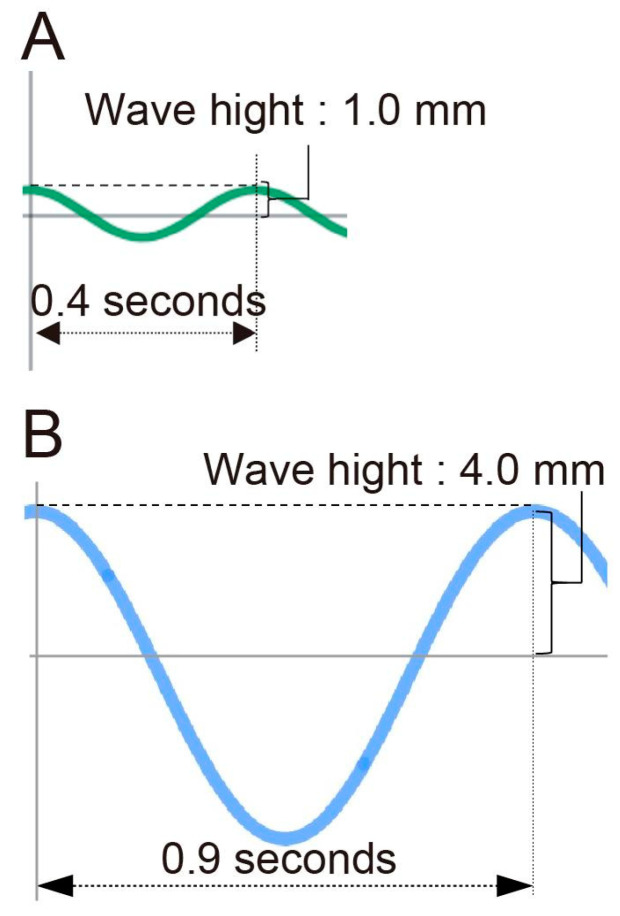
Measurement of Mechanical Stress. (**A**,**B**) The wave height and wave period measured in the unloaded group (**A**) and loaded group (**B**) under shaking.

We measured the height and frequency of waves occurring on the culture medium surface by shaking during organ culture to quantify the mechanical stress. In the unloaded group, the wave height was 1.0 mm and the frequency was 150 times/min, corresponding to a mechanical stress of 13.8 kcal. In contrast, in the loaded group, the wave height was 4.0 mm and the frequency was 67 times/min, corresponding to a mechanical stress of 496.0 kcal (Figure 2A,B). These values were selected to lie within a physiologically plausible range of loading, which, when converted into equivalent maternal physical activity, corresponds to moderate exercise. Furthermore, when a higher level of mechanical stress was applied to the loaded group, palatal shelf fusion was impaired in all samples.

### 3.2. Changes in YAP Expression in Palatal Tissue Induced by Mechanical Stress

**Figure 3:**
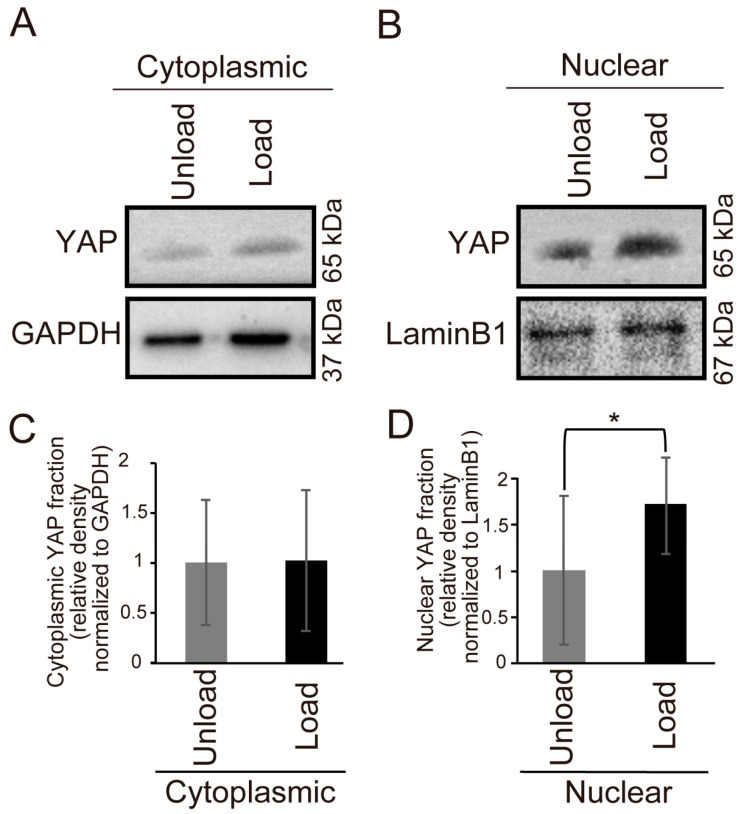
Comparison of YAP Expression in Mouse Fetal Palates Under Mechanical Stress. The expression of YAP in the palates of E13.5 mouse embryos after 24 h of organ culture was compared using Western blotting. (**A**,**B**) Western blot results. (**C**,**D**) Quantitative graphs of the corresponding results (* *p* < 0.05, error bars represent SD, *n* = 5 for each group). (**A**,**C**) Results for the cytoplasmic fraction. (**B**,**D**) Results for the nuclear fraction.

We compared YAP expression in the palatal tissues of 13.5-day-old mouse embryos after 24 h of organ culture using Western blot analysis. The results showed a significant increase in nuclear YAP expression in the loaded group compared to the unloaded group (Figure 3A–D).

### 3.3. Effects of Mechanical Stress on Palatal Tissue Growth

**Figure 4:**
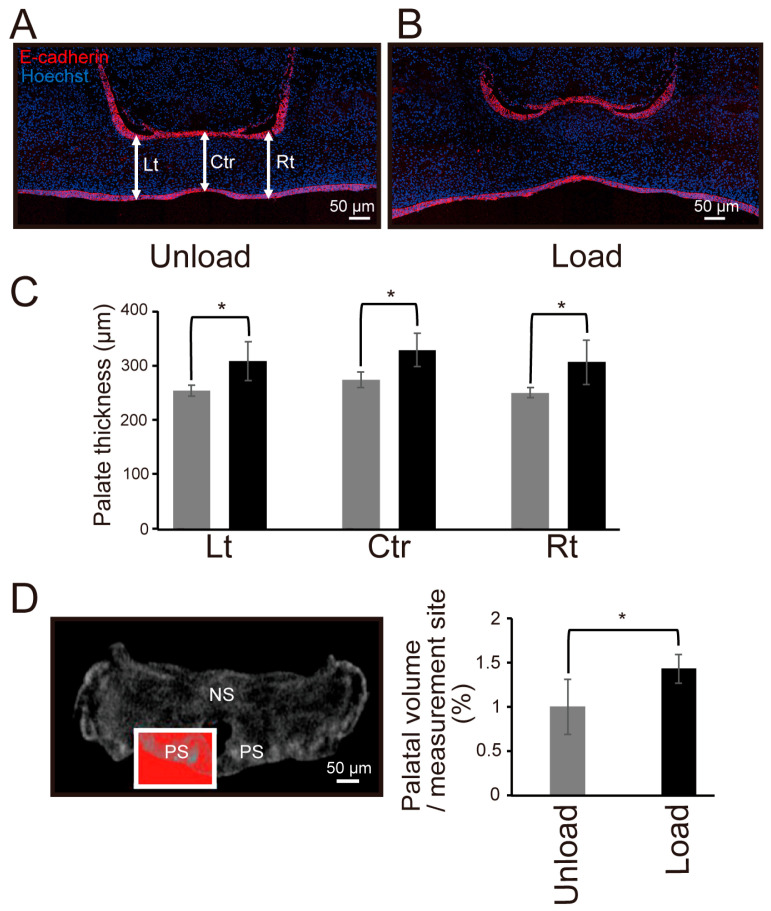
Comparison of Thickness and Volume of Mouse Fetal Palates Under Mechanical Stress. (**A**,**B**) Immunofluorescence staining image in a frontal section of the palatal tissue of an E14.5 mouse embryo after 24 h of organ culture. E-cadherin (red) marks the epithelial layer, and nuclei were counterstained with Hoechst (blue). (**A**) indicates the unloaded group, and (**B**) indicates the loaded group. The vertical thickness of the palatal process at three points: the left narrowest part (Lt), the fusion site (Ctr), and the right narrowest part (Rt), indicated by white arrows, was measured. (**C**) Measurement results of the vertical thickness of the palatal process at each measurement site. (* *p* < 0.05, error bars represent SD, *n* = 4 for each group) (**D**) Micro CT image of a frontal section of an E14.5 mouse embryo after 24 h of organ culture. The region enclosed by the red box was defined as the ROI, and the volume ratio (%) of the X-ray–opaque area relative to the ROI volume was calculated and compared. (NS: nasal septum, PS: palatal process, * *p* < 0.05, error bars represent SD, *n* = 3 for each group). Three-dimensional reconstruction was performed using CT Analyser software, and the X-ray opaque area within the defined ROI across 88 consecutive slices was quantified. The summed value of these areas was used as the volume index for comparison between groups.

We compared the vertical thickness of palatal shelves between the unloaded and loaded groups in organ-cultured palates from 14.5-day-old mouse embryos (Figure 4A,B). The results showed that the thickness of palatal shelves was significantly greater in the loaded group than in the unloaded group at all measured sites (Figure 4C). Additionally, micro-CT analysis revealed a significantly larger palatal shelf volume in the loaded group compared to the unloaded group (Figure 4D).

### 3.4. Effects of Mechanical Stress on Cell Proliferation and Death in the Palate

**Figure 5:**
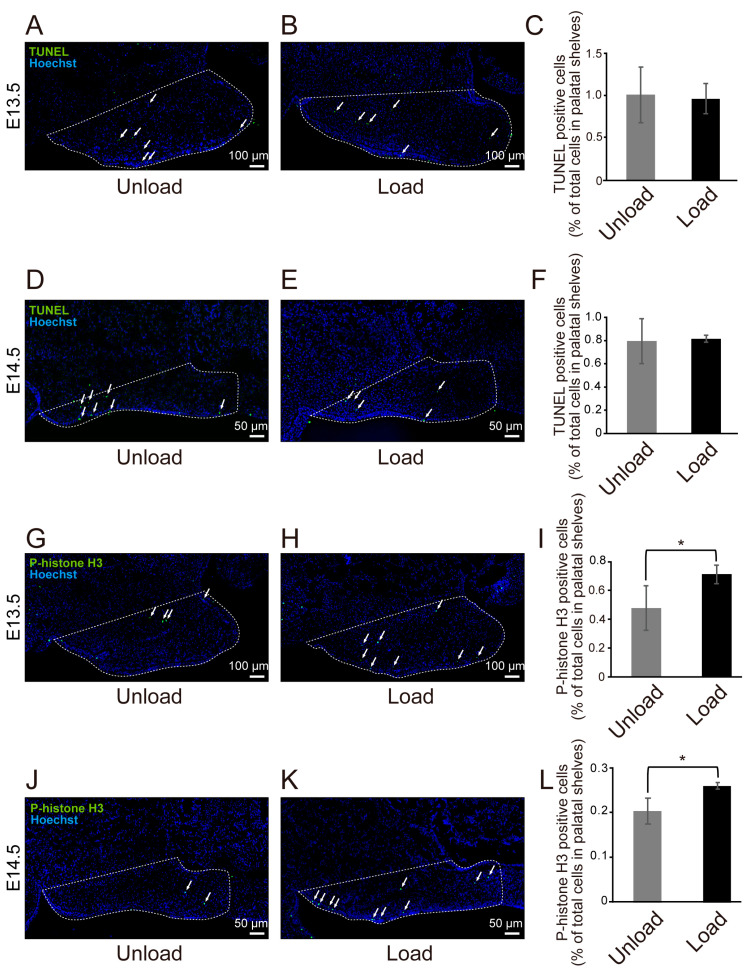
Comparison of Immunofluorescence Staining Results of Mouse Fetal Palatal Tissue Under Mechanical Stress. White arrows indicate representative TUNEL-positive or phospho-histone H3–positive cells. The dotted line outlines the palatal shelf region analyzed. (**A**,**B**) Representative images of TUNEL staining in right-side sections of the palatal tissue from E13.5 mouse embryos after 24 h of organ culture. Quantitative analyses were performed on both sides. The unloaded group is shown in (**A**) and the loaded group in (**B**). (**C**) Quantification of TUNEL-positive cells at E13.5 (* *p* < 0.05, error bars represent SD, *n* = 4 per group). (**D**,**E**) Representative images of TUNEL staining in right-side sections of the palatal tissue from E14.5 mouse embryos. The unloaded group is shown in (**D**) and the loaded group in (**E**). (**F**) Quantification of TUNEL-positive cells at E14.5 (* *p* < 0.05, error bars represent SD, *n* = 3 per group). (**G**,**H**) Representative images of phospho-histone H3 staining in right-side sections of the palatal tissue from E13.5 mouse embryos. The unloaded group is shown in (**G**) and the loaded group in (**H**). (**I**) Quantification of phospho-histone H3–positive cells at E13.5 (* *p* < 0.05, error bars represent SD, *n* = 5 per group). (**J**,**K**) Representative images of phospho-histone H3 staining in right-side sections of the palatal tissue from E14.5 mouse embryos. The unloaded group is shown in (**J**) and the loaded group in (**K**). (**L**) Quantification of phospho-histone H3–positive cells at E14.5 (* *p* < 0.05, error bars represent SD, *n* = 3 per group).

To examine the effects of mechanical stress on cell proliferation and death, we performed TUNEL staining and immunohistochemistry for phospho-histone H3 in palatal tissues from 13.5 and 14.5-day-old mouse embryos after 24 h of organ culture (Figure 5A–L). The results showed no significant difference in the percentage of TUNEL-positive cells between the unloaded and loaded groups at both E13.5 and E14.5 (Figure 5C,F). However, the percentage of phospho-histone H3-positive cells was significantly higher in the loaded group at both E13.5 and E14.5, indicating increased cell proliferation (Figure 5I,L).

### 3.5. Effects of Mechanical Stress on β-Catenin Expression

**Figure 6:**
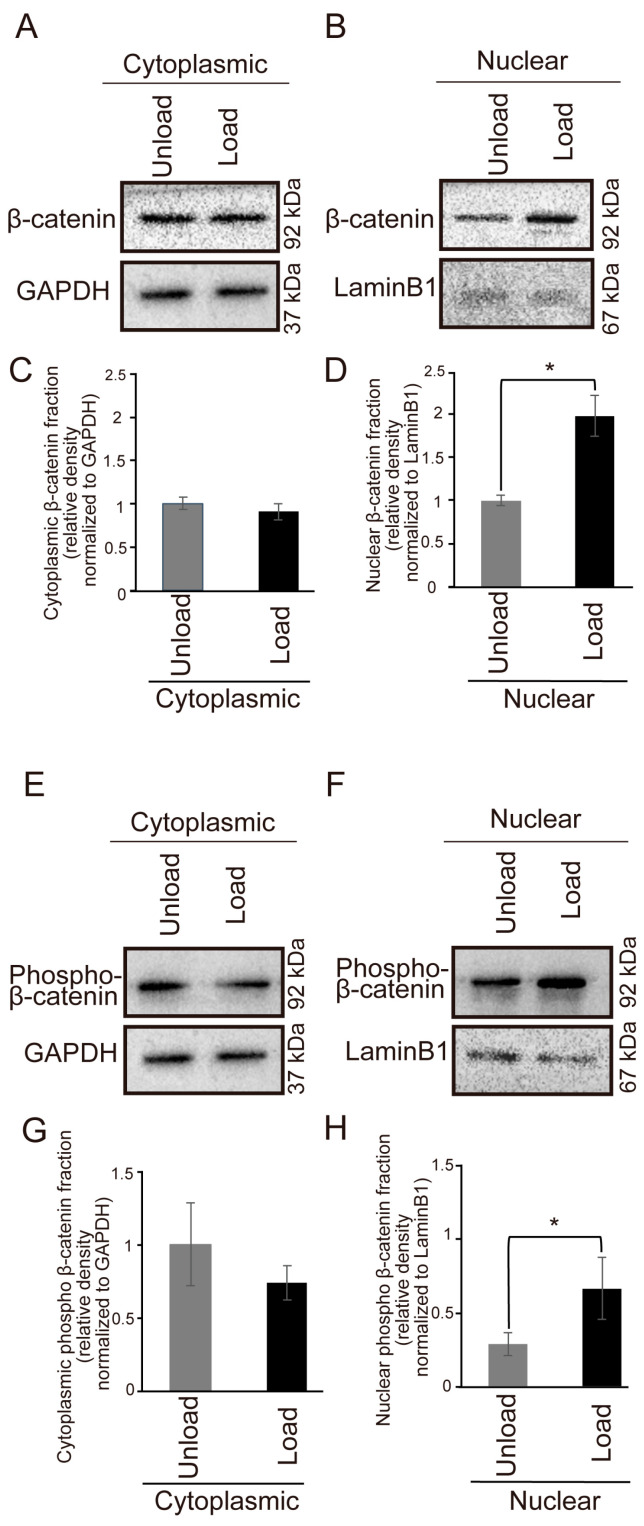
Changes in β-catenin Expression in Mouse Fetal Palatal Tissue Under Mechanical Stress. (**A**–**D**) Comparison of β-catenin expression in the palate of E13.5 mouse embryo after 24 h of organ culture. (**A**,**B**) Western blot results. (**C**,**D**) Quantitative graphs of the corresponding results. (**A**,**C**) Results for the cytoplasmic fraction. (**B**,**D**) Results for the nuclear fraction. (* *p* < 0.05, error bars represent SD, *n* = 5 for each group) (**E**–**H**) Comparison of phospho-β-catenin expression in the palate of an E13.5 mouse embryo after 24 h of organ culture. (**E**,**F**) Western blot results. (**G**,**H**) Quantitative graphs of the corresponding results. (**E**,**G**) Results for the cytoplasmic fraction. (**F**,**H**) Results for the nuclear fraction. (* *p* < 0.05, error bars represent SD, *n* = 7).

Western blot analysis revealed that nuclear translocation of β-catenin was significantly increased in the loaded group compared to the unloaded group (Figure 6A–D). Furthermore, Western blot analysis showed a significant increase in nuclear translocation of phospho-β-catenin in the loaded group (Figure 6E–H), suggesting activation of the Wnt/β-catenin signaling pathway.

### 3.6. Effects of Mechanical Stress on Osteogenic Differentiation

**Figure 7:**
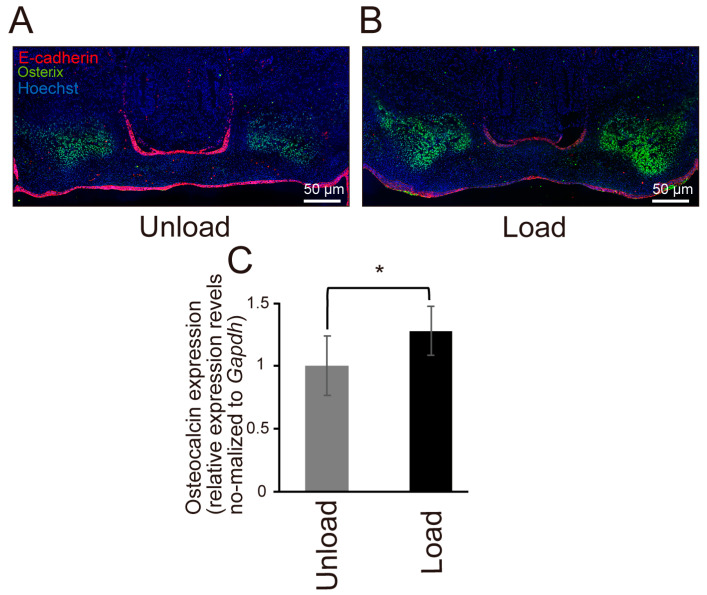
Comparison of Osteogenic Potential of Mouse Fetal Palatal Tissue Under Mechanical Stress. E-cadherin is shown in red, Osterix in green, and nuclei are counterstained with Hoechst (blue). (**A**,**B**) Immunofluorescence staining image of Osterix in a frontal section of an E14.5 mouse embryo after 24 h of organ culture. The unloaded group is shown in (**A**) and the loaded group is in (**B**). (**C**) Results of quantitative RT-PCR for Osteocalcin in the palate of an E14.5 mouse embryo after 24 h of organ culture. (* *p* < 0.05, error bars represent SD, *n* = 6 for each group).

Immunohistochemical staining for Osterix and quantitative RT-PCR analysis of Osteocalcin expression revealed that both osteogenic markers were upregulated in the loaded group (Figure 7A–C). The region showing marked Osterix expression in Figure 7A,B, likely corresponds to the alveolar process of the maxilla adjacent to the palate. These findings suggest that mechanical stress promotes early osteogenic differentiation–related signaling in the maxillary region, although complete ossification of the palatal bone was not observed within the 24-h culture period.

## 4. Discussion

The palate is formed through a series of processes including vertical growth of palatal shelves, elevation above the tongue, medial extension, and fusion at the midline, followed by three-dimensional growth. Although numerous studies have investigated palatal development, relatively few have focused on the three-dimensional growth following palatal shelf fusion. Accumulating evidence indicates that mechanical forces play important roles in craniofacial morphogenesis, including palatal development [8,9]. In particular, endogenous mechanical cues, such as cytoskeletal tension and extracellular matrix remodeling, have been shown to regulate palatal morphogenesis during fetal development [20]. In contrast, the effects of exogenous mechanical stimuli on palatal development remain poorly understood. While previous studies examining skeletal tissues have mainly employed stretching or compressive loading as forms of exogenous mechanical stress [28,29], no previous studies have applied oscillatory mechanical stimulation to palatal tissue during organ culture. In this study, we applied exogenous fluidic flow mechanical stress using wave energy and examined the effects on palatal development in organ-cultured mouse embryonic palates for 24 h. This culture method was designed to approximate, rather than fully reproduce, the mechanical forces that the fetal palate may experience in vivo. By simulating the mechanical stress experienced by the fetus in vivo, we successfully applied exogenous mechanical stress to palatal tissues under floating culture conditions. Epidemiological studies have suggested that reduced maternal physical activity is associated with an increased incidence of low birth weight and a higher risk of orofacial clefts [13,14]; however, the underlying biological mechanisms have remained largely unclear. In this study, we experimentally demonstrated for the first time that exposure of the fetus to appropriate levels of mechanical stimulation may contribute to normal palatal development. This finding implies that maternal movement and physical activity may play a supportive role in fetal craniofacial development, providing an important perspective that links maternal lifestyle, the mechanical environment experienced by the fetus, and the process of palatogenesis. Importantly, in the present study, the magnitude of exogenous mechanical loading was deliberately set within a physiologically plausible range that, when converted into equivalent maternal physical activity, corresponds to moderate exercise during pregnancy rather than excessive exertion. Our ex vivo culture method does not fully reproduce the in vivo mechanical conditions, but attempts to approximate the forces that the fetal palate may experience. Future studies could explore these effects in controlled in vivo settings. Furthermore, we calculated wave energy based on wave height and frequency, and confirmed that the unloaded group received 13.8 kcal and the loaded group received 496.0 kcal of mechanical stress (Figure 2A,B). When converted to human energy consumption, this corresponds to 4.6 min and 2.8 h of walking per day for the unloaded and loaded groups, respectively, indicating that the applied mechanical stress was not excessive.

To investigate the effects of exogenous mechanical stress on palatal shelf growth, we compared the vertical thickness and volume of palatal shelves at three sites between the unloaded and loaded groups. The results showed that the thickness of palatal shelves increased at all measured sites in the loaded group, and the volume was significantly larger (Figure 4A–D). These results suggest that mechanical stress may promote palatal growth regardless of the site, and that cell death may have decreased or cell proliferation may have increased in the loaded group compared to the unloaded group. To test this hypothesis, we compared the number of cells exhibiting cell proliferation and cell death. The results showed that cell proliferation was significantly promoted in the loaded group (Figure 5A–F), suggesting that mechanical stress promotes palatal shelf growth by stimulating cell proliferation.

To confirm that cells within the tissue were affected by mechanical stress, we examined YAP expression and found nuclear translocation in the loaded group (Figure 3A–D). This suggests that mechanical stress activates the Hippo pathway, leading to nuclear translocation of YAP. However, the cytoplasmic YAP expression did not change significantly in this study. When the Hippo pathway is activated, cytoplasmic YAP expression is expected to decrease due to nuclear translocation. However, the overall YAP expression in cells may have increased due to mechanical stress, resulting in no significant change in cytoplasmic YAP expression.

To explore the mechanism underlying cell proliferation induced by mechanical stress, we also examined the relationship between β-catenin expression and mechanical stress. The results showed nuclear translocation of β-catenin in the loaded group. β-catenin is a major mediator of the canonical Wnt/β-catenin pathway, and its nuclear translocation is a hallmark of the activation of this signaling pathway. Therefore, these results suggest the possibility of Wnt/β-catenin pathway activation by mechanical stress. Previous studies have reported that mechanical stress applied to human induced pluripotent stem cells (hiPSCs) increases β-catenin expression [30] and that nuclear-localized YAP is involved in stabilizing β-catenin in the nucleus and activating transcription of β-catenin target genes [31]. Based on these findings, we suggest that nuclear-localized YAP induced by mechanical stress may activate transcription by β-catenin, thereby promoting cell proliferation in our study. Although we did not assess RNA expression of YAP- or β-catenin-related genes in this study, future analyses at the transcriptional level may provide additional mechanistic insight into how exogenous mechanical stress regulates palatal development.

Furthermore, another study reported that β-catenin is expressed in the epithelial covering of the palatal shelves and that its suppression prevents the fusion of the left and right shelves, leading to cleft palate [6]. This suggests that in cases of cleft palate caused by decreased β-catenin expression in the epithelium, mechanical stress could potentially improve palatal fusion by promoting β-catenin expression. Although we did not focus on the epithelial-specific expression of β-catenin in this study, the results suggest that exogenous mechanical stress may regulate β-catenin expression. Therefore, future studies focusing on β-catenin expression in the epithelium and the relationship between mechanical stress and palatal shelf fusion are needed to further understand palatal development and cleft palate formation. In addition, we attempted to evaluate the elevation of palatal shelves at E13.5 after organ culture. However, because the palatal shelves at this stage are composed mainly of soft tissue, they were easily deformed before fixation with paraformaldehyde, which made it technically difficult to obtain reliable measurements. Further methodological improvements will be required to assess palatal shelf elevation under in vitro culture conditions.

β-catenin has also been reported to be involved in osteogenic differentiation in addition to cell proliferation [32]. Therefore, we examined the effects of mechanical stress on osteoblast differentiation. The results showed a significant increase in Osterix expression in the loaded group compared to the unloaded group, suggesting that mechanical stress may also affect osteoblast differentiation. Previous studies have reported that Wnt7b is involved in osteoblast differentiation downstream of Indian hedgehog (Ihh) [33] and that Wnt ligands in the canonical Wnt/β-catenin pathway induce the expression of Runx2, which is essential for osteoblast differentiation [24]. β-catenin is a common effector of the Hippo pathway and the canonical Wnt/β-catenin pathway, suggesting that the interaction between these pathways may play an important role in palatal development and warrants further investigation.

## 5. Conclusions

Our findings suggest that exogenous mechanical stress promotes cell proliferation and palatal development through activation of the Hippo pathway and subsequent nuclear translocation of β-catenin. Additionally, our results indicate that mechanical stress may also influence osteogenic differentiation in the palate.

## Figures and Tables

**Table 1:** Primer sequences used in this study.

Primer	Direction	Sequence
Gapdh	forward	5′-CATCACTGCCACCCAGAAGACT-3′
	reverse	5′-ATGCCAGTGAGCTTCCCGTTCA-3′
Osteocalcin	forward	5′-GCAATAAGGTAGTGAACAGACT-3′
	reverse	5′-CCATAGATGCGTTTGTAGGCGG-3′

## Data Availability

The data supporting the findings of this study are available from the corresponding author upon reasonable request.
